# Factors regulating the gripping force and stiffness of 25- and 27-gauge internal limiting membrane forceps

**DOI:** 10.1371/journal.pone.0310419

**Published:** 2024-11-05

**Authors:** Kana Katakami, Hisanori Imai, Yasuyuki Sotani, Hiroko Yamada, Makoto Nakamura

**Affiliations:** 1 Division of Ophthalmology, Department of Surgery, Kobe University Graduate School of Medicine, Kobe, Japan; 2 Department of Ophthalmology, Kansai Medical University, Hirakata, Japan; Akita University: Akita Daigaku, JAPAN

## Abstract

This study aimed to identify the factors affecting the gripping force and stiffness of 25-gauge and 27-gauge (25G and 27G, respectively) internal limiting membrane (ILM) forceps and to compare the effect of these factors on various ILM forceps manufactured by different companies. This study evaluated 25G and 27G ILM forceps with two different types of tip shapes, Eckardt and Maxgrip, manufactured by Alcon (A), DORC (B), VitreQ (C), and Katalyst (D). The gripping force was defined as the force required to move the ILM forceps away from a thin paper by pulling the paper. Shaft stiffness was determined by measuring the shaft displacement under a known force. Multiple regression analysis revealed that the gripping force showed significant correlations with the gauge (P<0.001), type of shaft tip (Eckardt/Maxgrip) (P<0.001), and contact area of the tip (P<0.001). The shaft stiffness showed significant correlations with the gauge (P<0.001), length of the base (P<0.001), thickness of the metal of the shaft (P = 0.05), and lumen area of the shaft (P = 0.01). The gripping force and shaft stiffness differed for each product. Thus, vitreoretinal surgeons must select the appropriate type of ILM forceps based on their characteristics.

## Introduction

Vitrectomy using a one-port pars plana approach, first performed by Machemer in 1972, marked a significant advancement in the field of vitreous surgery [1]. Fundus observation was performed under microscopic illumination in the past; however, the intensity of microscopic illumination is insufficient to facilitate in-depth examination [1]. This led to the introduction of light guides. Light sources have transitioned from halogens to xenon, mercury lamps, and light-emitting diodes over the years [2, 3]. Chandelier endo-illumination was also introduced to facilitate adequate illumination of the surgical fields [2, 3]. The transition of vitreous cutter from a drill-driven to a guillotine-type instrument has vastly improved the safety of vitreous removal [4, 5]. In addition, this transition has also led to a significant increase in the rotation speed and suction pressure of the cutter, thereby facilitating finer vitreous removal and reduced fluttering in the vitreous body and retina, further enhancing safety. The introduction of bi-blade cutters has also dramatically improved the safety and efficiency of vitreous removal [5]. Beveled-tip cutters, which bring the cutting edge of the cutter closer to the retina, have been used for the segmentation and delamination of the fibrovascular proliferative membrane. The versatile use of vitreous cutters—for instance, as substitutes for spatulas—has become more common [6, 7]. In parallel with these advancements, there has been remarkable progress in minimizing the invasiveness of vitreous surgery via refinement of the incision size. Specifically, vitreous surgery, which initially started with a 17-gauge one-port approach, now involves 27-gauge three-port surgery [8], thereby reducing the requirement for placing sutures at the port site at the end of the surgery. The sutureless nature of these small incisions has contributed to a significant reduction in postoperative pain, discomfort, and erythema [9–11]. The use of small incisions in vitreous surgery has also reduced the incidence of postoperative astigmatism [12, 13]. Thus, advancements in basic tools and minimization of surgical incisions have refined and simplified vitreous surgery.

However, downsizing instruments such as vitreous cutters, light guides, backflush needles, and internal limiting membrane (ILM) forceps for smaller incisions is challenging because of complications arising from the reduction in the thickness of the instruments [14–17]. Various innovations have aided in overcoming these limitations associated with vitreous cutters, such as the decrease in the efficiency of vitreous removal and the stiffness of the shaft [18–23]. However, the problems associated with peripheral instruments, including the backflush needle and ILM forceps, remain unresolved [16]. Clinicians must be aware of the features of each product when considering which product to use, as the performance varies significantly based on the products used. Reporting these aspects may contribute to improved surgical outcomes.

Therefore, this study aimed to compare and evaluate the performance of ILM forceps, a crucial and frequently used peripheral instrument, to provide insights into their features.

## Materials and methods

Approval from an Ethics Committee was not required as this was a non-clinical study. ILM forceps of two gauges, 25- and 27-gauge (25G and 27G, respectively), were evaluated. The forceps were also distinguished by the tip shape: Eckardt and Maxgrip type (Ek and Mx, respectively). Forceps manufactured by the following companies were evaluated in this study (Table 1): Company A, 25+ and 27+ ^™^ REVOLUTION DSP ILM Forceps (Ek) and 25+^™^ and 27+^™^ GRIESHABER MAXGrip^™^ Forceps REFLEX DSP (Mx) (Alcon Grieshaber AG; Schaffhausen, Switzerland); Company B, Eckardt End-gripping 25G and 27G (Ek) and Ultra peel 25G and 27G (Mx) (DORC. International; Zuidland, The Netherlands); Company C, Eckardt Endgripping (Ek) 25G and 27G Forceps and Shah Xtra Grip (Mx) 25G and 27G Forceps (VitreQ BV; Vierpolders, The Netherlands); and Company D, 25G DEX^™^ Maculorhexis Forceps (Ek) and 27G DEX^™^ Stiff Maculorhexis Forceps (Ek) and 25G DEX^™^ Super grip (Mx) and 27G DEX^™^ Stiff Super grip (Mx) (Katalyst Surgical, LLC; Chesterfield, MO, US).

**Table 1:** List of forceps types and manufacturers.

Company		Size of gauge	Eckardt (Ek)	Maxgrip (Mx)
A	Alcon	25G	REVOLUTION DSP ILM Forceps	GRIESHABER MAXGrip^™^ Forceps REFLEX DSP
		27G		
B	DORC	25G	Eckardt Endgripping	Ultra peel
		27G		
C	VitreQ	25G	Eckardt Endgripping	Shah Xtra Grip
		27G		
D	Katalyst	25G	DEX^™^ Maculorhexis Forceps	DEX^™^ Super grip
		27G	DEX^™^ Stiff Maculorhexis Forceps	DEX^™^ Stiff Super grip

The following parameters were included in the analysis: gauge, type of shaft tip (Ek/Mx), gripping force, displacement of the shaft, length of the base, length of the shaft, lumen area of the shaft, metal thickness of the shaft, and contact area of the tip.

**Fig 1:**
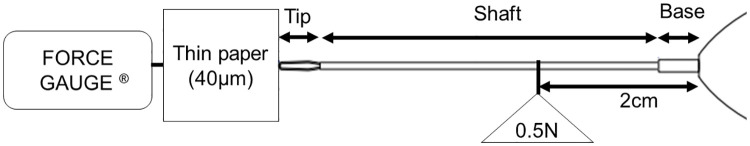
Schematic representation depicting the evaluation of the gripping force and shaft stiffness of the ILM forceps. The gripping force was measured by pulling a 40-μm thin paper with each ILM forceps. The stiffness of the ILM forceps was evaluated by measuring shaft displacement under a force of 0.5 N at 20 mm from the shaft base. Shaft stiffness is proportional to the added force and cube of the distance from the base to the point of force, whereas it is inversely proportional to Young’s modulus and the moment of inertia of area. ILM, internal limiting membrane.

The gripping force, defined as the maximum pulling force, was measured using a digital force gauge (FORCE GAUGE; SATO SHOUJI INC., Kanagawa, Japan). Grip force was measured by pulling a 40-μm thin paper with each ILM forceps (Fig 1).

The stiffness of the shaft of the forceps, a measure of the overall bendability of the metals constituting the shaft, was evaluated by measuring the shaft displacement under a force of 0.5 N 20 mm from the shaft base, as described previously [23]. The shaft stiffness is proportional to the added force and cube of the distance from the base to the point of force (Fig 1), whereas it is inversely proportional to Young’s modulus and the moment of inertia of area. The force and distance from the base to the point of force were maintained at constant values in this study, as described above. A greater deflection indicates that the material is more likely to bend as Young’s modulus and the moment of inertia of the area are numerical values related to the material and shape of the material constituting the shaft. The displacement of the shaft was measured using a digital caliper (Shinwa Digital NOGISU; Shinwa Rules Co., Ltd., Niigata, Japan). Five sets of each ILM forceps were prepared, and the shaft stiffness of each was measured and used in the analysis.

**Fig 2:**
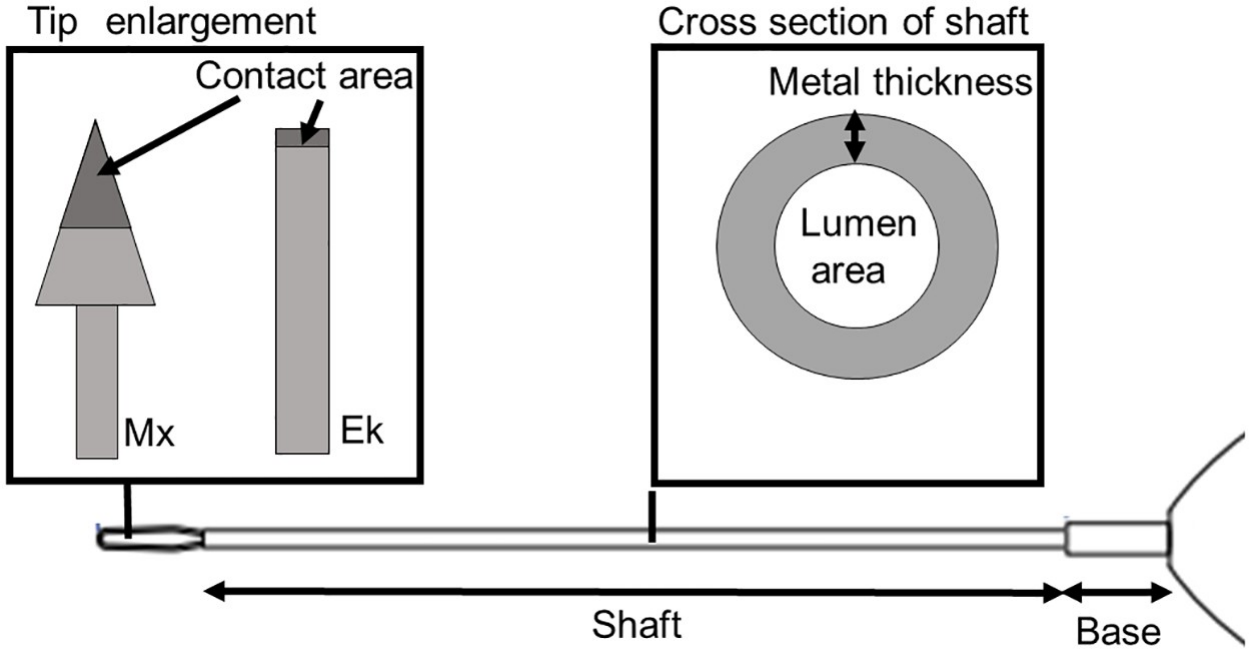
Schematic of the structure of the ILM forceps. For the tip, the type of shaft tip (Ek or Mx) and the width of the contact area were measured. For the shaft, the lengths of the shaft and base, as well as the external and internal diameters of the shaft, were measured to determine the metal thickness and the lumen area. ILM, internal limiting membrane.

The shaft of each product was first disassembled to measure the length of the base, length of the shaft, lumen area, metal thickness, and contact area of the tip. The lengths of the disassembled shafts were measured using a digital caliper (Shinwa Digital NOGISU; Shinwa Rules Co., Ltd., Niigata, Japan) with an accuracy of 0.1 mm. The measured value was defined as the “full length of the shaft.” The disassembled shafts and tips were positioned vertically under a smartphone camera equipped with a microscope (Nurugo Micro Smartphone Microscope; Nurugo) that captured images at 400× magnification. The lumen area of the shaft, metal thickness of the shaft, and contact area of the tip were determined using image analysis software (version 23.5.1, Adobe Photoshop; Adobe Systems, San Jose, California, USA). All parameters for the five sets of forceps were measured and used in the analysis (Fig 2).

### Statistical methods

The gripping force, shaft stiffness, and all parameters of the shaft were compared using the Kruskal–Wallis *H*-test, followed by a post hoc analysis using the Mann–Whitney *U* test with Bonferroni correction. Significant correlations between all parameters of the shaft and the gripping force and shaft stiffness were analyzed using Spearman’s correlation analysis.

The parameters contributing to the gripping force and the shaft stiffness were identified via multiple regression analysis. All statistical analyses were performed using SPSS software (version 24.0; SPSS Inc., Chicago, IL, USA). A P-value of <0.05 was considered statistically significant.

## Results

Table 2 presents a comprehensive overview of the basic data pertaining to the ILM forceps. Comprehensive analysis of the ILM forceps manufactured by multiple companies revealed distinct patterns for the gripping force and shaft stiffness of the 25G and 27G forceps.

**Table 2:** Basic details of the internal limiting membrane forceps.

Size of gauge	Company	A (Alcon)		B (DORC)		C (VitreQ)		D (Katalyst)	
	Forceps type (Ek/Mx)	Eckardt	Maxgrip	Eckardt	Ultrapeel	Eckardt	Shar Xtra Grip	Stiff Maculorhexis	Super grip
25G	Gripping force (g)	19.4±3.4	155.0±5.4	120.6±16.5	142.9±15.2	58.7±5.4	109.1±6.6	70.6±11.5	114.3±23.6
	Shaft displacement (mm)	3.1±0.1	2.7±0.2	2.9±0.1	3.0±0	2.3±0.3	2.6±0.2	2.9±0.1	2.9±0.1
	Base length(mm)	2.5±0	3.2±0	3.2±0	3.2±0	3.7±0	3.7±0	0±0	0±0
	Shaft length (mm)	27.5±0	28.2±0	32.2±0	32.2±0	32.0±0	32.0±0	30.1±0	30.1±0
	Lumen area of shaft (mm^2^)	0.1321±0.0071	0.1098±0.0029	0.1333±0.0048	0.1346±0.0067	0.1281±0.0031	0.1281±0.0031	0.0856±0.0066	0.0952±0.0063
	Metal thickness of shaft (mm)	0.0450±0.0055	0.0630±0.0024	0.0440±0.0037	0.0430±0.0051	0.0480±0.0024	0.0480±0.0024	0.0850±0.0063	0.0760±0.0058
	Contact area of tip (mm^2^)	0.0258±0.0032	0.2489±0.0103	0.0283±0.0031	0.2649±0.0312	0.0369±0.0087	0.2392±0.0285	0.0126±0.0010	0.1811±0.0077
27G	Gripping force (g)	31.6±3.5	175.2±3.7	29.7±6.0	90.6±13.0	42±4.0	54.8±8.5	75.4±13.3	94.7±17.3
	Shaft displacement (mm)	6.9±0.2	4.4±0.2	7.5±0.6	4.04±0.1	3.8±0.2	3.8±0.2	1±0	1±0
	Base length (mm)	2.5±0	3.2±0	3.2±0	3.2±0	3.7±0	3.7±0	13±0	13±0
	Shaft length (mm)	27.5±0	28.2±0	29.7±0	28.5±0	32±0	32±0	31±0	31±0
	Lumen area of shaft (mm^2^)	0.0581±0.0017	0.0700±0.0041	0.0834±0.0025	0.0688±0.0023	0.0784±0.0024	0.0814±0.0038	0.0625±0.0052	0.0590±0.0044
	Metal thickness of shaft (mm)	0.0640±0.0020	0.0497±0.0042	0.0370±0.0024	0.0520±0.0024	0.0420±0.0024	0.0390±0.0037	0.0590±0.0058	0.0630±0.0051
	Contact area of tip (mm^2^)	0.0093±0.0013	0.1497±0.0141	0.0247±0.0020	0.2259±0.0218	0.0328±0.0031	0.2466±0.0197	0.0079±0.0011	0.2097±0.0136

**Fig 3:**
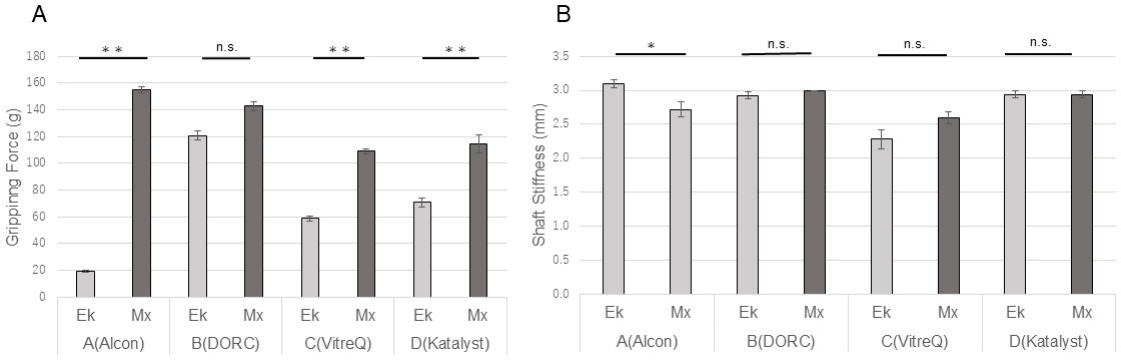
Comparisons of the gripping force and shaft stiffness among the 25G forceps. For Companies A, C, and D, the gripping force of the Mx forceps was significantly higher than that of the Ek forceps (**p<0.01). No significant difference was observed for Company B. For Company A, the shaft stiffness of the Ek forceps was significantly lower than that of the Mx forceps (*p<0.05). No significant difference was observed for Companies B, C, and D. Ek: Eckardt type, Mx: Maxgrip type.

When manufactured by Companies A, C, and D, the 25G Mx forceps showed a significantly higher gripping force than did the 25G Ek forceps. However, the 25G forceps manufactured by Company B showed no statistically significant difference in the gripping force (P<0.01) (Fig 3A). The shaft stiffness of the 25G Mx forceps was significantly higher than that of the 25G Ek forceps (P = 0.04) when these were manufactured by Company A. However, no significant differences were observed between these forceps when manufactured by companies B, C, and D (Fig 3B).

**Fig 4:**
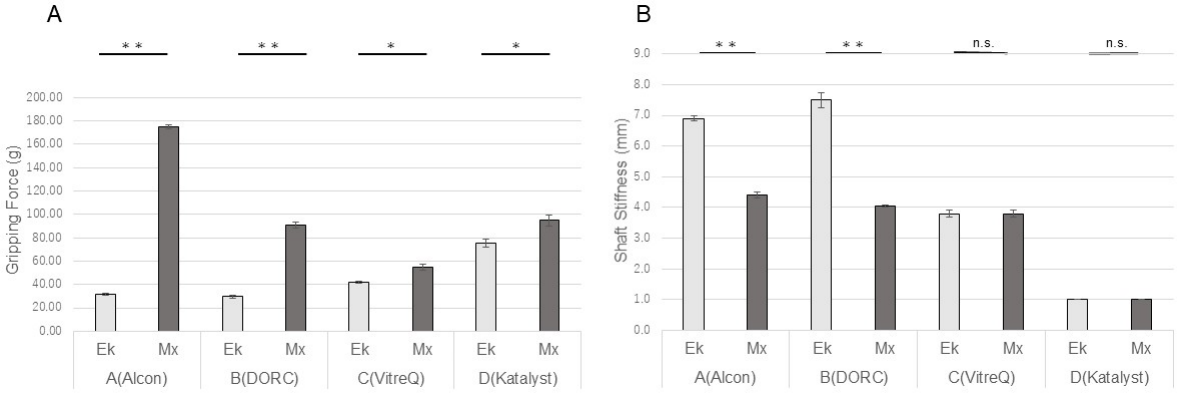
Comparisons of the gripping force and shaft stiffness among the 27G forceps. The gripping force of the Mx forceps was significantly higher than that of the Ek forceps from all companies (**p<0.01, *p<0.05). For Companies A and B, the shaft stiffness of the Ek forceps was significantly lower than that of the Mx forceps (**p<0.01). No significant difference was observed for Companies C and D. Ek: Eckardt type, Mx: Maxgrip type.

The gripping force of the 27G Mx forceps was significantly higher than that of the 27G Ek forceps manufactured by all companies (Fig 4A). The shaft stiffness of the 27G Ek forceps was significantly higher than that of the 27G Mx forceps when these were manufactured by Companies A and B; however, no significant difference was observed between these forceps when they were manufactured by companies C and D (Fig 4B).

**Fig 5:**
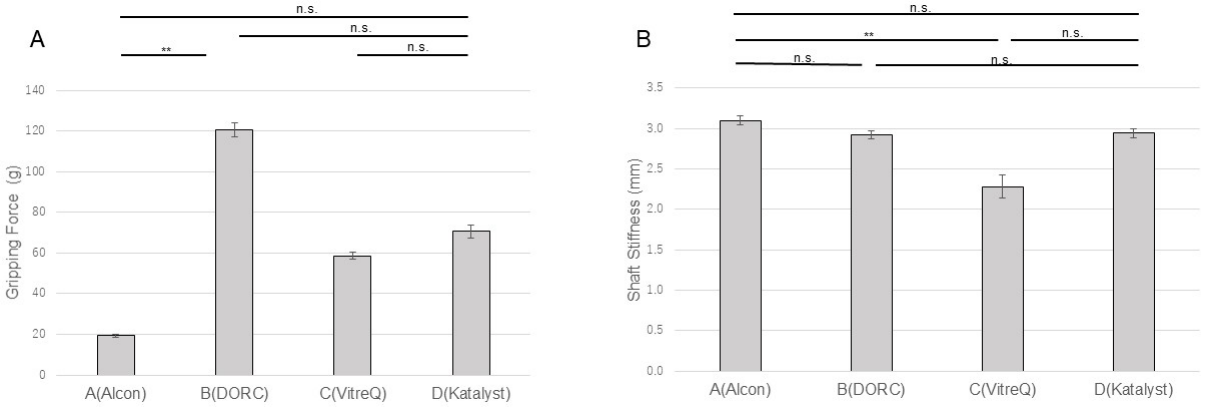
Comparisons of the gripping force and shaft stiffness among the 25G Ek forceps from different companies. (A) The gripping force of the forceps manufactured by Company B was significantly higher than that of the forceps manufactured by Company A (P<0.01). (B) The shaft stiffness of the forceps from Company C was greater than that of the forceps from Company A (P<0.01). Ek: Eckardt type.

**Fig 6:**
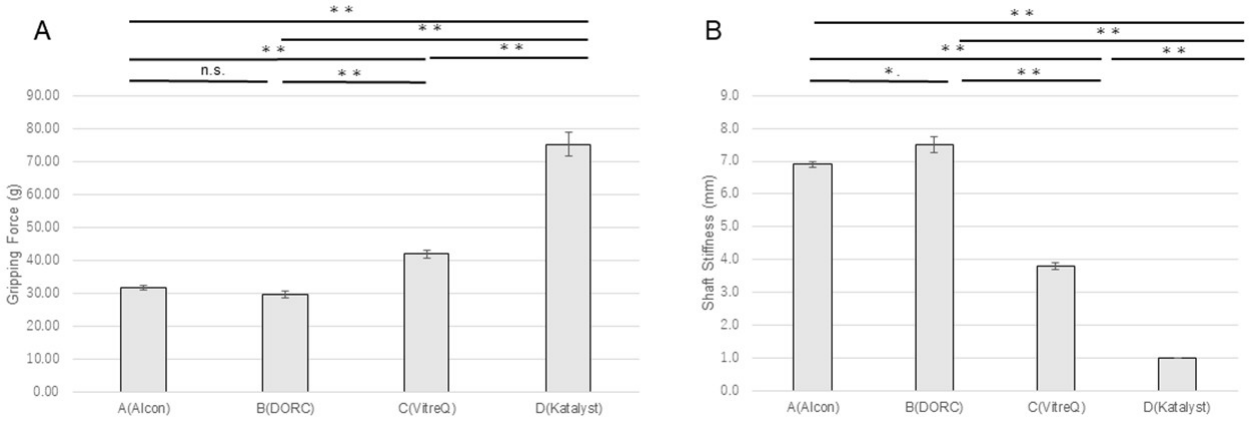
Comparisons of the gripping force and shaft stiffness among the 27G Ek forceps from different companies. The gripping force and shaft stiffness of the forceps manufactured by Company D were the highest. The gripping force and shaft stiffness of the forceps manufactured by Company C were both higher than those of the forceps manufactured by Companies A and B (P<0.01). Ek: Eckardt type.

The potential of the products was compared using the tip shape as an evaluation factor. Further investigation of the 25G forceps revealed that the gripping force of the Ek forceps manufactured by Company B was significantly higher than that of the Ek forceps manufactured by Company A (P<0.01) (Fig 5A). The shaft stiffness of the Ek forceps manufactured by Company C was higher than that of the Ek forceps manufactured by Company A (P<0.01) (Fig 5B). Among the 27G forceps, the gripping force and shaft stiffness of the Ek forceps manufactured by Company D were the highest. The gripping force and shaft stiffness of the Ek forceps manufactured by Company C were higher than those of the Ek forceps manufactured by Companies A and B (P<0.01 for both) (Fig 6A and 6B).

**Fig 7:**
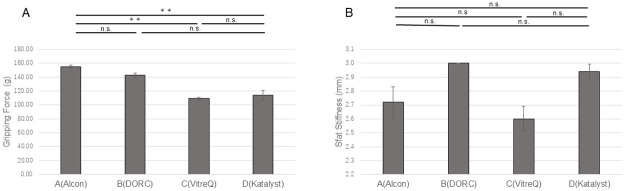
Comparisons of the gripping force and shaft stiffness among the 25G Mx forceps from different companies. (A) The forceps from Company A had a significantly higher gripping force than did the forceps from Companies C and D (P<0.01). (B) No significant differences in the shaft stiffness were observed among forceps from different companies. Mx: Maxgrip type.

**Fig 8:**
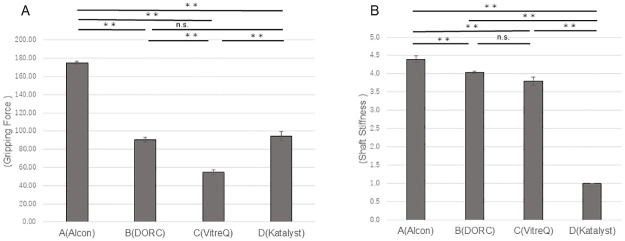
Comparisons of the gripping force and shaft stiffness among the 27G Mx forceps from different companies. (A) The forceps from Company A had the highest gripping force among forceps from all companies (P<0.01), whereas the forceps from Company C exhibiting the lowest gripping force (P<0.01). (B) The forceps from Company D had the highest shaft stiffness among forceps from all companies (P<0.01), whereas the forceps from Company A had the lowest shaft stiffness (P<0.01). Mx: Maxgrip type.

When comparing the Mx forceps among all companies, for the 25G type, a higher gripping force was observed for the forceps from Company A than for the forceps from Companies C and D (P<0.01) (Fig 7A). No significant differences in the shaft stiffness were observed among forceps from the different companies (Fig 7B). For the 27G forceps, the gripping force of the Mx forceps from Company A was the highest (P<0.01), whereas that of the forceps from Company C was the lowest (P<0.01). The Mx forceps from Company D had the highest shaft stiffness (P<0.01), whereas that from Company A had the lowest shaft stiffness (P<0.01) (Fig 8A and 8B).

Multiple regression analysis was conducted using gripping force as the dependent variable. The factors that were correlated with the gripping force were used as the independent variables in the analysis. The size of the gauge, type of shaft tip, and contact area of the tip were identified as significant determinants (Table 3). An additional separate multiple regression analysis was performed with the shaft stiffness as the dependent variable. An item that demonstrated a correlation with the shaft stiffness was used as the independent variable. The gauge, length of the base, lumen area of the shaft, and metal thickness of the shaft were identified as significant factors (Table 4).

**Table 3:** Multiple regression analysis with gripping force as an independent variable.

	Correlation		Multiple regression		
	r	*p* value	β	SE	*p* value
Size of gauge (27G/25G)	0.26	0.02	0.32	7.36	<0.01
Type of shaft tip (Eckardt/Maxgrip)	0.68	<0.01	1.49	25.14	<0.01
Contact area of the tip(mm^2^)	0.55	<0.01	-2.07	121.55	<0.01

Multiple regression analysis including gripping force as an independent variable. r, Pearson correlation coefficient; *β*, partial correlation coefficient; SE, standard error

**Table 4:** Multiple regression analysis with shaft stiffness an independent variable.

	Correlation		Multiple regression		
	r	*p* value	β	SE	*p* value
Size of gauge (27G/25G)	-0.439	<0.01	-2.03	1.77	<0.01
Base length (mm)	-0.89	<0.01	-0.87	0.03	<0.01
Lumen area of shaft (mm^2^)	-0.19	0.02	1.37	32.05	0.013
Metal thickness of shaft (mm)	-0.36	<0.01	0.58	34.17	0.048

Multiple regression analysis including shaft stiffness an independent variable. r, Pearson correlation coefficient; *β*, partial correlation coefficient; SE, standard error

Comparison of the contact area of the tip revealed that the contact area of the tip of the Mx forceps was significantly larger than that of the Ek forceps for both gauges (P<0.01). The contact area of the tip of the 25G Ek forceps was significantly larger than that of the 27G Ek forceps (P = 0.04). However, the contact area of the tip of the 25G Mx forceps did not differ significantly from that of the 27G Mx forceps (P = 0.09) (Table 5).

**Table 5:** Statistical comparison of the contact area of the tip.

	Contact area of tip (mm^2^)		*p* value
	25G	27G	
Ek	0.0259±0.0103	0.0187±0.0110	0.04
Mx	0.2335±0.0396	0.2079±0.0412	0.09
*p* value	<0.01	<0.01	

**Fig 9:**
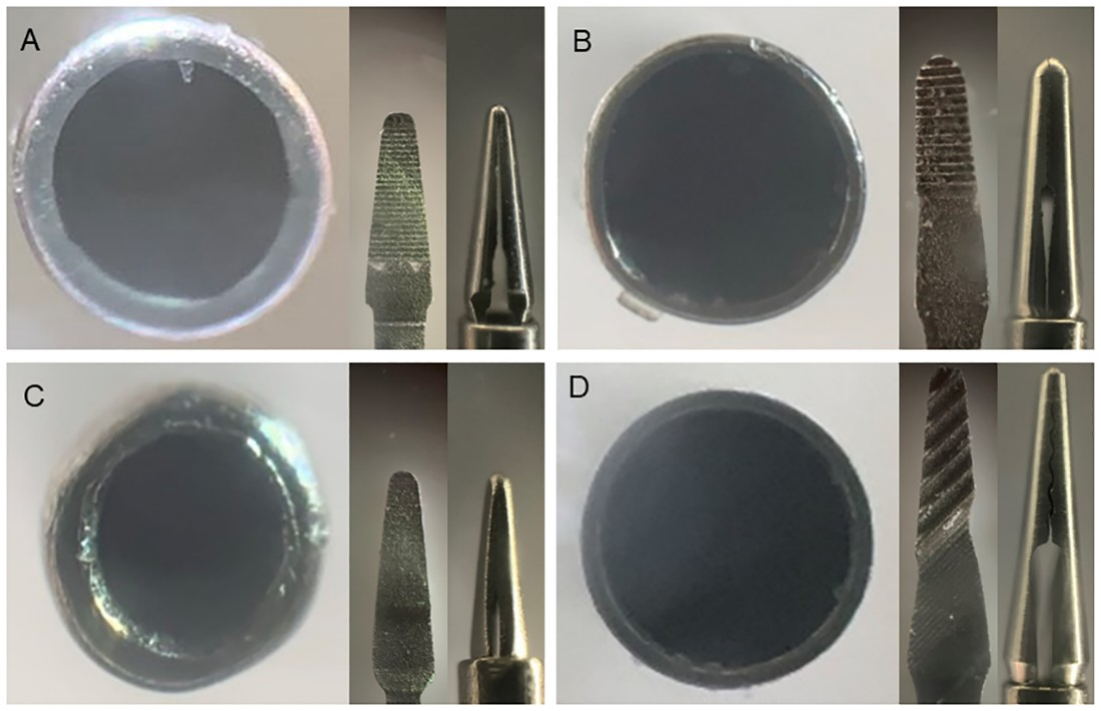
Comparisons among 25G Mx forceps from different companies. Magnified photographs showing the cross-sections of the shafts and contact areas of the tips of the 25G Mx forceps, as viewed from the front and the side for forceps from each company. The metal thickness of the shaft and the structure of the contact area vary among forceps from different companies (A: Alcon; B: DORC; C: Katalyst; D: Vitre Q). Mx: Maxgrip type.

**Fig 10:**
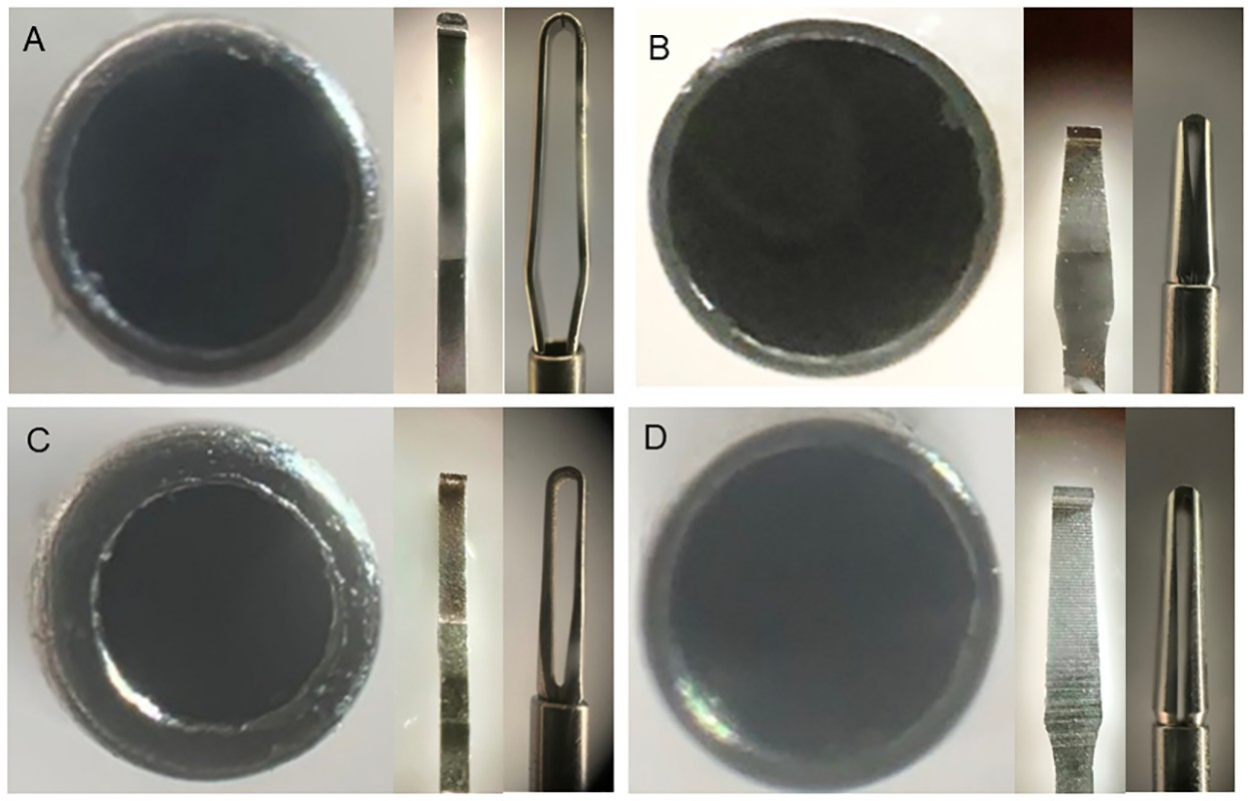
Comparisons among 25G Ek forceps from different companies. Magnified photographs showing the cross-sections of the shafts and contact areas of the tips of the 25G Ek forceps, as viewed from the front and the side for forceps from each company. The metal thickness of the shaft and the structure of the contact area vary among forceps from different companies (A: Alcon; B: DORC; C: Katalyst; D: Vitre Q). Ek: Eckardt type.

**Fig 11:**
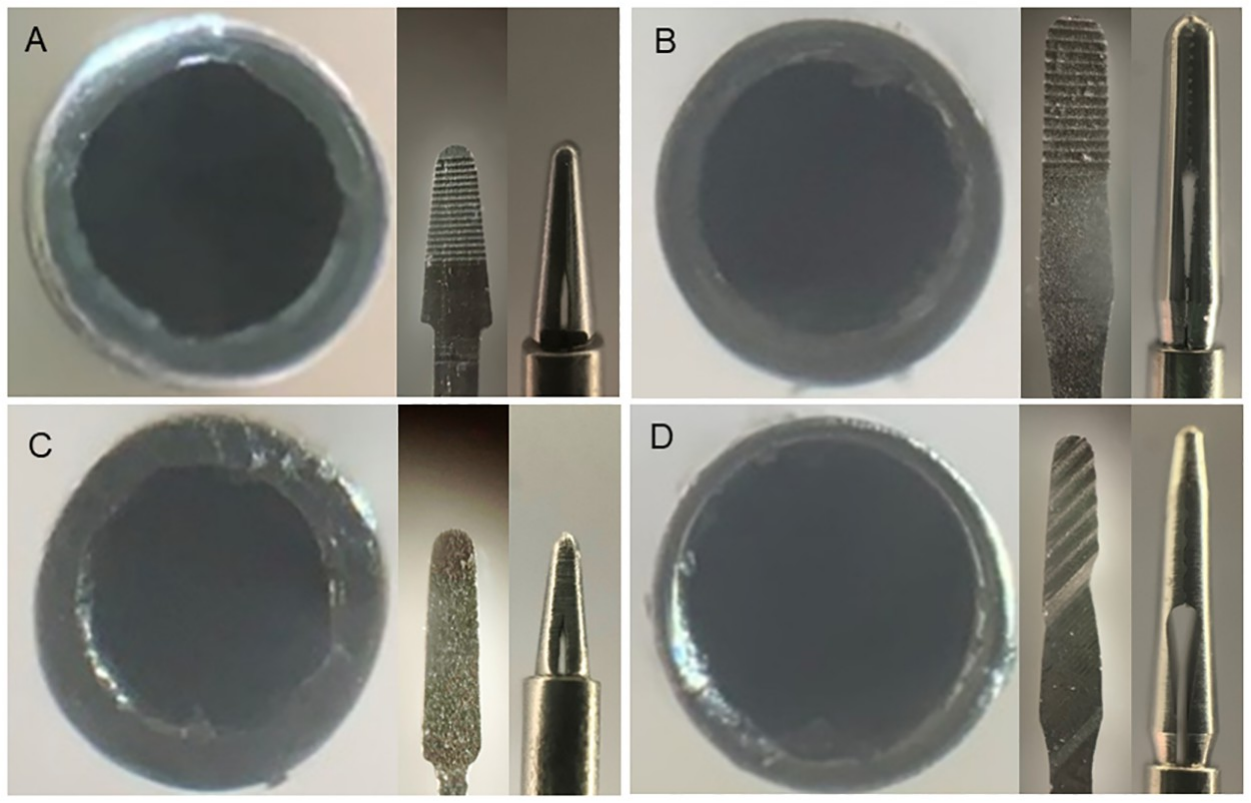
Comparisons among 27G Mx forceps from different companies. Magnified photographs showing the cross-sections of the shafts and the contact areas of the tips of the 27G Mx forceps, as viewed from the front and the side for forceps from each company. The metal thickness of the shaft and the structure of the contact area vary among forceps from different companies (A: Alcon; B: DORC; C: Katalyst; D: Vitre Q). Mx: Maxgrip type.

**Fig 12:**
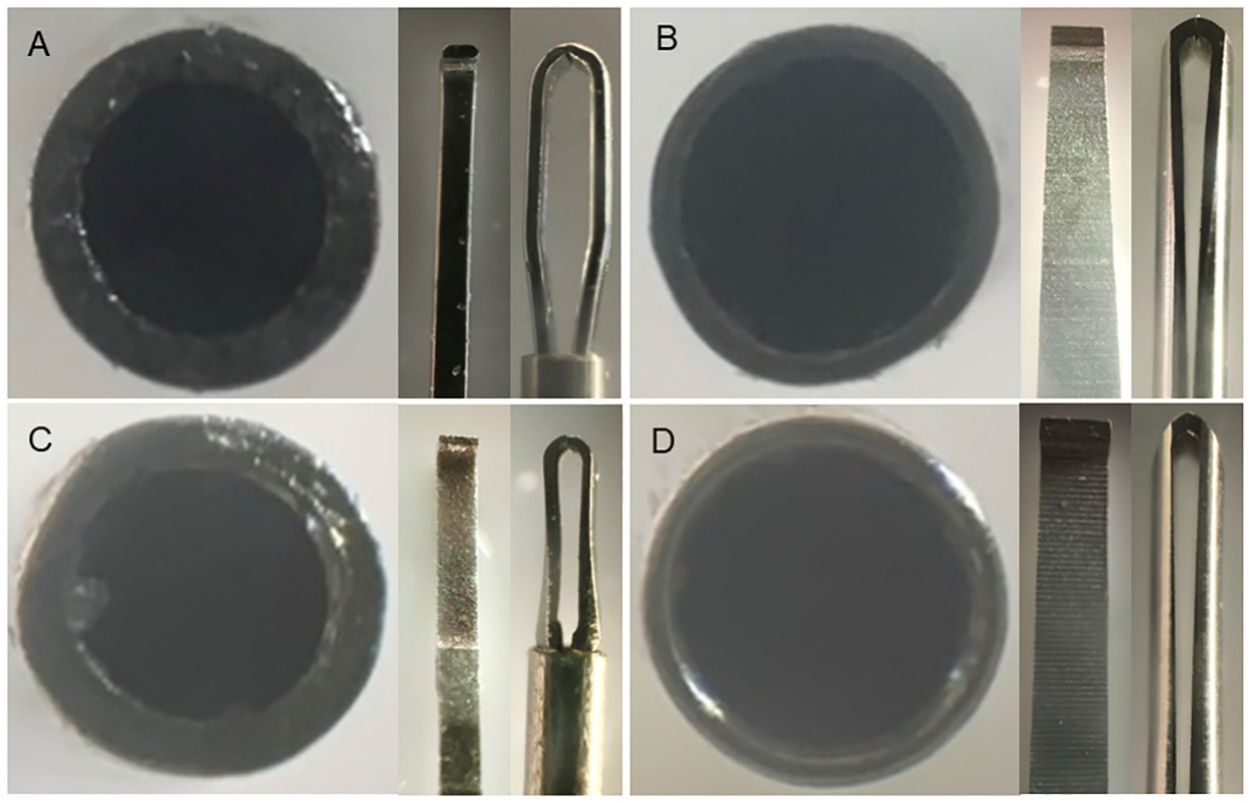
Comparisons among 27G Ek forceps from different companies. Magnified photographs showing the cross-sections of the shafts and the contact areas of the tips of the 27G Ek forceps, as viewed from the front and the side for forceps from each company. The metal thickness of the shaft and the structure of the contact area vary among forceps from different companies (A: Alcon; B: DORC; C: Katalyst; D: Vitre Q). Ek: Eckardt type.

Comprehensive analysis of all products revealed variations among the forceps manufactured by the companies in two key aspects: the metal thickness of the shaft and the structure of the contact area. Table 2 and Figs 9–12 present the details of these differences. In addition, the designs of the tips of the Ek and Mx forceps exhibited distinct differences in the present study. The comparative morphology of these tips underscores the variability in the designs adopted by different companies.

## Discussion

ILM forceps are tools used to grasp and peel the ILM involved in various retinal vitreous diseases, such as macular holes, vitreomacular traction syndrome, macular edema, and epiretinal membrane. The thickness of ILM ranges between 0.3 and 1.5 μm [24]. However, the ILM may adhere strongly to the retinal tissue in some conditions [25]. The removal of ILM requires precision and delicacy owing to the thin and variable nature of this tissue. In addition, peeling of the proliferative membrane, which is thicker than ILM, is also necessary in patients with epiretinal membrane, proliferative diabetic retinopathy, and proliferative vitreoretinopathy. ILM forceps require a significant gripping force as these membranes are strongly adhered to the retinal tissue. Therefore, ILM forceps play a crucial role in vitreous surgery and require a balance of various elements such as gripping force and the shaft stiffness.

The findings of the present study indicate that forceps that exhibit both a high gripping force and high shaft stiffness are not available at present. Thus, researchers and clinicians must aim to develop improved ILM forceps. Moreover, users must assess the performance of the various ILM forceps available and identify the products suitable for specific retinal diseases and conditions. This underscores the importance of making informed choices when selecting the appropriate tools for different clinical conditions.

Multivariate analysis using gripping force as the dependent variable revealed that the gauge, type of shaft tip, and contact area of the tip were significant factors. The contact area for Ek forceps was significantly wider for 25G when the tip shape was identical; however, the Mx forceps showed no difference between the gauges. In contrast, the gripping area of the Mx forceps was significantly larger than that of the Ek forceps for the same gauge. These findings suggest that it may be more suitable to select a 25G system for diseases requiring a higher gripping force or involving proliferative membranes strongly adherent to the retinal surface. Moreover, use of the Mx forceps may enhance the efficiency of membrane peeling.

Multivariate analysis using the shaft stiffness as the dependent variable revealed that the gauge, length of the base, metal thickness of the shaft, and lumen area of the shaft as significant factors. The shaft stiffness plays a crucial role in facilitating stable surgical operations. A certain instrument length is necessary in the case of eyes with long axial length, such as the eyes of individuals with high myopia, narrow palpebral fissures, and a high nasal bridge. Therefore, selecting a longer 25G system, which yields sufficient shaft stiffness even at a certain length, may be preferable. However, achieving a balance using any instrument remains a challenge and an avenue for future investigation.

Examination of the tip shapes using high-magnification photographs revealed notable findings. The Mx forceps had a rounded contact area with the retina owing to its arc-shaped tip regardless of the gauge. In contrast, the Ek forceps had a rectangular corner as the contact area with the retina owing to its rectangular tip. This raises concerns regarding the potential for retinal damage unless the forceps contact the retina vertically. Insights into these areas may aid vitreoretinal surgeons in selecting appropriate instruments, ensuring safer surgeries, and providing better visual outcomes for patients.

The inability to compare and examine all types of forceps are limitations of this study. Nevertheless, the information obtained in this study regarding the aspects of the devices affecting the gripping force and shaft stiffness is considered highly valuable. As various products continue to be manufactured and sold, the results of this study serve as fundamental information for considering the gripping force and shaft stiffness of any product.

In conclusion, the characteristics of ILM forceps vary significantly among manufacturers and tip shapes. Vitreous surgeons should be familiar with the features of these instruments and select them carefully to improve surgical outcomes.
